# Marked differences in tight junction composition and macromolecular permeability among different intestinal cell types

**DOI:** 10.1186/s12915-018-0481-z

**Published:** 2018-02-01

**Authors:** Sarah C. Pearce, Arwa Al-Jawadi, Kunihiro Kishida, Shiyan Yu, Madeleine Hu, Luke F. Fritzky, Karen L. Edelblum, Nan Gao, Ronaldo P. Ferraris

**Affiliations:** 1 Department of Pharmacology, Physiology and Neurosciences, New Jersey Medical School, Rutgers University, Newark, NJ 07103 USA; 2 Present address: Performance Nutrition Team, Combat Feeding Directorate, US Army, 15 General Greene Ave, Natick, MA 01760-5018 USA; 3 Present address: Department of Science and Technology on Food Safety, Kindai University, Wakayama, 649-6493 Japan; 4 Department of Biological Sciences, Rutgers University, Life Science Center, 225 University Avenue, Newark, NJ 07102 USA; 5 Department of Pathology & Laboratory Medicine, Center for Inflammation and Immunity, Rutgers New Jersey Medical School, Newark, NJ 07103 USA; 6 Advanced Microscopic Imaging Core Facility, New Jersey Medical School, Rutgers University, Newark, NJ 07103 USA

**Keywords:** Claudin, Differentiation, Enterocyte, Epithelia, Leak pathway, Paracellular, Permeability, Small intestine, Stem cells, Tight junction

## Abstract

**Background:**

Mammalian small intestinal tight junctions (TJ) link epithelial cells to one another and function as a permselective barrier, strictly modulating the passage of ions and macromolecules through the pore and leak pathways, respectively, thereby preventing the absorption of harmful compounds and microbes while allowing regulated transport of nutrients and electrolytes. Small intestinal epithelial permeability is ascribed primarily to the properties of TJs between adjoining enterocytes (ENTs), because there is almost no information on TJ composition and the paracellular permeability of nonenterocyte cell types that constitute a small but significant fraction of the intestinal epithelia.

**Results:**

Here we directed murine intestinal crypts to form specialized organoids highly enriched in intestinal stem cells (ISCs), absorptive ENTs, secretory goblet cells, or Paneth cells. The morphological and morphometric characteristics of these cells in organoids were similar to those in vivo. The expression of certain TJ proteins varied with cell type: occludin and tricellulin levels were high in both ISCs and Paneth cells, while claudin-1, -2, and -7 expression was greatest in Paneth cells, ISCs, and ENTs, respectively. In contrast, the distribution of claudin-15, zonula occludens 1 (ZO-1), and E-cadherin was relatively homogeneous. E-cadherin and claudin-7 marked mainly the basolateral membrane, while claudin-2, ZO-1, and occludin resided in the apical membrane. Remarkably, organoids enriched in ENTs or goblet cells were over threefold more permeable to 4 and 10 kDa dextran compared to those containing stem and Paneth cells. The TJ-regulator larazotide prevented the approximately tenfold increases in dextran flux induced by the TJ-disrupter AT1002 into organoids of different cell types, indicating that this ZO toxin nonselectively increases permeability. Forced dedifferentiation of mature ENTs results in the reacquisition of ISC-like characteristics in TJ composition and dextran permeability, suggesting that the post-differentiation properties of TJs are not hardwired.

**Conclusions:**

Differentiation of adult intestinal stem cells into mature secretory and absorptive cell types causes marked, but potentially reversible, changes in TJ composition, resulting in enhanced macromolecular permeability of the TJ leak pathway between ENTs and between goblet cells. This work advances our understanding of how cell differentiation affects the paracellular pathway of epithelia.

**Electronic supplementary material:**

The online version of this article (10.1186/s12915-018-0481-z) contains supplementary material, which is available to authorized users.

## Background

The main functions of the mammalian small intestine are to digest and absorb nutrients as well as to act as a permselective barrier preventing the entry of harmful molecules and microbes while still allowing the selective passage of dietary nutrients, ions, and water. The small intestinal cells contain digestive enzymes and transporters that process nutrients and electrolytes, but the barrier between cells is essential because the lumen contains large amounts of bacteria, viruses, food allergens, toxins, and excreted metabolic byproducts, as well as varying concentrations of ions and solutes that can be markedly different from those of internal fluids. This barrier, which is permselectively sealed by tight junctions (TJs), has at least two types of pathways: the high-capacity pore pathway regulating mainly the transport of ions and small molecules, and the low-capacity leak pathway modulating the flux of large uncharged macromolecules [1, 2]. There is a general consensus that claudins regulate mainly the pore pathway, mediating the paracellular flux of ions as well as small molecules up to ~0.8 nm in diameter, and that zonula occludens (ZO-1 or *Tjp1*), occludin (OCLN, *Ocln*), and tricellulin (*Marveld2*) regulate the leak pathway, mediating the flux of macromolecules up to ~6 nm [2–5]. The relationship between the leak and pore pathways is poorly understood [6]. Thus, TJ proteins are essential components of gut epithelia and must work in concert with one another to maintain physiological homeostasis in animals. Adherens junctions and desmosomes are also found between cells but these exhibit mainly structural functions.

Numerous studies on TJ proteins have focused primarily on their function in enterocytes (ENTs), the cell type that constitutes most of the small intestinal epithelium. There is little known, however, about TJ function and TJ protein distribution in nonenterocyte cell types of the gut mucosa. This is understandable, because ENTs constitute ~80% of small intestinal mucosal epithelia, and most transformed cell lines used for studies of human intestinal diseases, like Caco-2, have been selected for their ENT-like characteristics [7, 8]. Thus, small intestinal paracellular permeability is ascribed primarily to properties of specific TJs between adjoining ENTs. However, there are large numbers of nonenterocytes lining the intestinal villi: mucus-secreting goblet cells (GOBs, comprising ~5–10% of the total cell population), hormone-secreting enteroendocrine cells (~1%), and chemosensory tuft cells (< 1%), which are derived from secretory progenitors [9–12]. Secretory Paneth cells (PANs, < 5% of cells) produce antimicrobial peptides as well as growth factors that maintain the neighboring intestinal stem cells (ISCs, < 1%) in the crypt region.

Since ~20% of the intestinal epithelium comprises nonenterocytes, then their potential contributions to the permeability of the pore and leak paracellular pathways affecting intestinal and whole-body homeostasis are critical. Gastrointestinal distress arising from food allergies, inflammatory bowel diseases, pathogenic bacteria, and viruses as well as gut dysbiosis typically results in marked perturbations in intestinal paracellular permeability. Since there is little information about the TJ composition in nonenterocytes, it is not clear whether their TJs are more vulnerable to these insults than ENT TJs. Genetic diseases caused by mutations in TJ proteins [2, 13, 14] can affect some cell types more than others. Specifically, the extracellular part of TJ proteins associated with a certain cell type can participate in homophilic (homotypic) *trans* interactions with TJs of the same cell type, or in heterophilic interactions with different TJs of similar or different cell types, determining in large part the paracellular permeability between these cells [15]. These potential interactions are difficult to characterize, as the TJ proteins associated with different nonenterocyte cells are mostly unknown.

Several TJ proteins, like claudin -2, -3, -4, -7, -10, and -15, involved in the TJ pore pathway are distributed heterogeneously along the crypt–villus axis [6, 16], suggesting that claudins that constitute the TJ between crypt-residing cells, like ISCs and PANs, may differ from those in villus-residing cells, like ENTs and GOBs. In particular, claudin-2, which constitutes the leaky and cation-selective paracellular channels of TJs, is found mainly in intestinal crypts, where it likely mediates cation permeabilities [16, 17]. In contrast, there is little information about the crypt–villus distribution of ZO-1, occludin, and tricellulin, which regulate the TJ leak pathway, and about the macromolecular permeability of the paracellular pathway among different cell types. Previous studies have provided mathematical estimates of the paracellular permeability along the crypt–villus axis, although these did not distinguish the pore from the leak pathway [18, 19]. Such predictions have not been tested experimentally, because the types and properties of TJs between intestinal cell types other than ENTs remain mostly unexplored.

In this study, we tested the hypotheses that TJ proteins are distributed heterogeneously among the various cell types that constitute the intestinal mucosa, and that the leak pathway differs between cell types that comprise the villus from those that are found in the crypt. To evaluate these hypotheses, we produced organoids enriched in ENTs (~90% of all cells), GOBs (~80%), PANs (~65%), or ISCs (~90%) [20, 21], then determined the expression of different TJ proteins and the permeability of 4 to 10 kDa dextran in these organoids. We also tested the hypothesies that TJ composition is characterized by developmental plasticity, by examining the paracellular attributes of ENT-enriched organoids when these are forcibly dedifferentiated to reacquire ISC-like properties, and that the TJ-disrupter AT1002 (an active fragment of the ZO toxin) and TJ-regulator larazotide modulate macromolecular permeability in organoids.

## Methods

### Animals

All procedures conducted in this study were approved by the Institutional Animal Care and Use Committee, New Jersey Medical School, Rutgers University. Crypts from the proximal intestine were isolated from 6–8-week-old mice (Taconic Laboratories, Hudson, NY). Intestinal organoids were isolated as previously described [20, 21].

### Directed differentiation of intestinal organoids

Based upon methods from previous work [20, 21], we directed differentiation of crypts into organoids enriched in ISCs, ENTs, GOBs, and PANs. Briefly, small molecules (Wnt and Notch activators/inhibitors) including CHIR99021 (3 μM; Stemgent, Cambridge, MA), 2 mM valproic acid (VPA; Tocris Bioscience, United Kingdom), 2 μM C59 (Stemgent, Cambridge, MA), and 10 μM DAPT (Tocris Bioscience, United Kingdom) were added to the base crypt culture medium (CCM) containing EGF (50 ng/ml; Life Technologies), murine R-spondin-1 (500 ng/mL; PeproTech), and murine Noggin (100 ng/mL; PeproTech). Organoids in CCM with all cell types represented are referred to as typical (TYP). ENT, GOB, and PAN organoids are differentiated from ISC organoids, and characterized at ~3 days after initiation of differentiation when biomarker expression becomes stable [20]. Bright-field images were obtained using an ECLIPSE TS100-F microscope (Nikon, Japan).

### Dedifferentiation

Crypts were isolated and differentiated into ENT organoids as described above. Briefly, after 72 h in ENT media, ENT organoids were dedifferentiated by adding ISC media containing 6 μM CHIR + 2 mM VPA for ~2 days [20] to produce dedifferentiated ENTs (dENTs).

### Real-time polymerase chain reaction

Total RNA was extracted from intestinal organoids using a commercially available kit (RNeasy Micro, Qiagen). Real-time polymerase chain reaction using Mx3000P (Stratagene, La Jolla, CA) was used to analyze cDNA using Maxima SYBR green (Thermo Fisher Scientific, Grand Island, NY). Primer sequences (Integrated DNA Technologies, Coralville, IA) are listed in Additional file 1: Table S1. All samples were standardized to β–actin expression.

### Staining

Organoids were fixed in 4% paraformaldehyde and gently spun down to a pellet (at 100 *g*). The supernatant was removed and organoids were resuspended in agarose gel and then embedded in paraffin. Paraffin-embedded organoids were then cut into 5-μm sections using a Leica RM2235 microtome (Leica Biosystems, Wetzlar, Germany), applied onto superfrost plus slides, and air dried at room temperature for 37 °C overnight. Samples were subsequently de-paraffinized then stained with hematoxylin and eosin (H&E) and periodic acid-Schiff (PAS)/Alcian blue, and immunocytochemistry was then performed. Antigen retrieval by 10 min of incubation in boiling 10 mM sodium citrate buffer (pH 6.0) was done for immunostained slides. Antibody information is presented in Additional file 2: Table S2. The morphometrics of sections stained with H&E or PAS was determined (Nikon Microphot FXA, Nikon Instruments Inc., Melville, NY) then analyzed using ImageJ (imagej.nih.gov; [20]) while immunofluorescence measurements of histological sections were recorded by confocal microscopy (Olympus Fluoview 1000, Olympus Corporation, Center Valley, PA).

### Permeability

Since our previous work [22] has demonstrated that dextran is suitable for determining intestinal permeability to macromolecules, we used 4 to 10 kDa fluorescein isothiocyanate (FITC)-labeled dextrans that have a Stokes radius of 1.3 to 2 nm [23] to characterize the steric properties of the leak pathway of organoids enriched in different cell types. It seems that 40 kDa dextrans having ~4.5 nm radius is impermeable to the TJs of typical organoids [24]. The paracellular permeability of organoids was tested using FITC-dextran (4 kDa and 10 kDa) [24]. Briefly, directed organoids were generated in individual Matrigel wells each housing TYP, ISC, ENT, GOB, and PAN organoids. Wells were washed twice with phosphate buffered saline (PBS; pH 7.4) and incubated for 30 min in 4 and 10 kDa FITC-dextran at a final concentration of 1.25 μM for 30 min at room temperature, to impose a chemical serosal-to-lumen gradient. We determined by fluorescence intensity the amount that would accumulate in the organoid lumen. After incubation, the FITC-dextran was removed and the organoids were gently washed five times with PBS to remove the background. Fluorescence within an organoid was estimated (ImageJ) by focusing on the entire luminal area inside the organoid. The representative background fluorescence was estimated from four different areas surrounding the organoid. The net fluorescence in an organoid is the integrated luminal fluorescence density less the mean fluorescence of background readings and the mean autofluorescence. The net fluorescence was then averaged from several representative organoids from different wells, and this mean fluorescence was normalized to that of a TYP organoid.

### Time course

Organoids were seeded in 50% Matrigel using an eight-well chambered cover glass (Fisher Scientific, Hampton, NH) for 7 days, then differentiated into ISC, ENT, GOB, and PAN organoids as described above. Three days after differentiation, organoids were imaged with a spinning disk confocal microscopy using an inverted DMi8 microscope (Leica Microsystems Inc., Buffalo Grove, IL) equipped with a CSU-W1 spinning disk and ZYLA SL150 sCMOS camera (Andor USA, Concord, MA), a 20×/0.40 CORR dry objective, and iQ3 acquisition software (Andor). Then, 5-mm Z-stacks were acquired, ranging 25 mm above and below the vertical midpoint of the organoid. A total of ten organoids were imaged per well and 1.25 μM 4 kDa FITC-dextran was then added to the media, which was allowed to incubate for 10, 30, and 60 min at 37 °C and 5% CO_2_. The 4 kDa FITC-dextran-containing media was removed, and wells were gently washed as described. Following washout, Z-stacks were acquired for the same organoids imaged prior to FITC-dextran addition.

### AT1002 and larazotide treatment

ENT and ISC organoids were challenged with 10 μg/mL AT1002, a six-mer active fragment of ZO toxin (LifeTein, Somerset NJ, USA; [25]), 12.5 mM larazotide acetate (INN-202, gift of J. Madan and S. Laumas, Innovate Biopharmaceuticals, Inc., Raleigh, NC, USA), or a combination of the two. AT1002 disrupts the epithelial barrier while larazotide acetate restores barrier function by rearrangement of actin [26, 27]. After an overnight incubation (37 °C) with AT1002 and/or larazotide, a 4 kDa dextran permeability assay was conducted following the protocol mentioned above.

### Statistical analyses

Data are presented as means ± standard error. To analyze the significance of organoid type, genotype, or organoid age, either a one-way or multi-way ANOVA was used with a Tukey test for multiple comparisons. Differences were considered significant at *P* ≤ 0.05 (StatView, Abacus Concepts, NC).

## Results

### Directed differentiation of small intestinal organoids

#### Cell histology and morphometrics

**Fig. 1:**
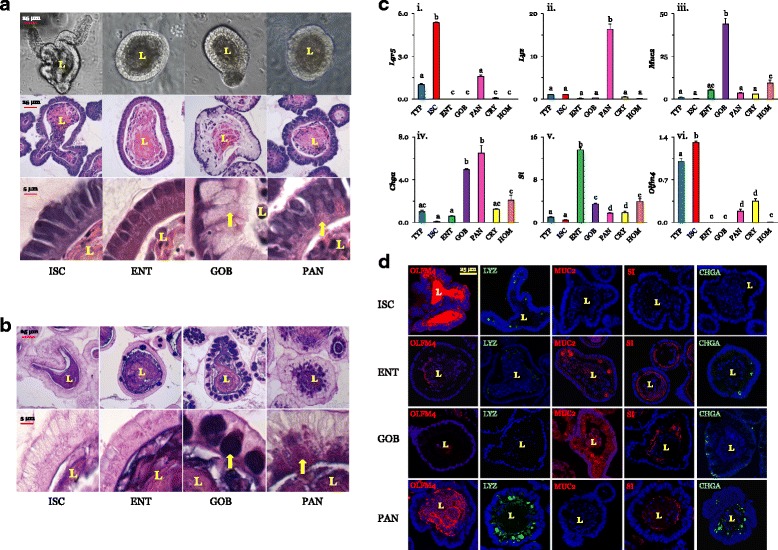
Bright-field and H&E images of ISC, ENT, GOB, and PAN organoids. **a** Images at 20× (top and middle) and 60× (bottom row). There were no differences in morphology of cells in two sets of organoids from different mice. Arrows depict secretory vesicles in GOBs and PANs. **b** Periodic acid-Schiff/Alcian blue stained images at 20× (top) and 60× (bottom). **c** Expression profile of biomarkers in TYP, ISC, ENT, GOB, PAN, intestinal crypts (CRY) and tissue homogenates (HOM). TYPs are undirected organoids with all cell types represented. The expression of each biomarker was normalized to that in TYP organoids (=1.0). **i** The biomarker for stem cells, leucine-rich-repeat-containing G-protein coupled receptor 5 (Lgr5), was highly expressed in ISC organoids (*n* = 4 for TYP and *n* = 3 for other organoids). **ii** PANs are marked by lysozyme (Lyz), which was very abundant in PAN organoids (see scale along ordinate axis) (*n* = 4 for TYP; *n* = 3 for others). **iii** Mucin-2 (Muc2) was highly expressed in GOBs (*n* = 4 for TYP and *n* = 3 for others). **iv** The enteroendocrine biomarker chromogranin A (Chga) was expressed in GOBs and PANs (*n* = 4 for TYP and *n* = 3 for others). **v** ENTs were marked by sucrase isomaltase (SI) (*n* = 4 for TYP, ISC, and HOM, and *n* = 3 for others). **vi** Stem cells were marked by olfactomedin-4 (*Olfm4*) (*n* = 4 for TYP, ENT; *n* = 3 for others). *P*^a,b,c,d^ ≤ 0.05. **d** Immunofluorescence of biomarkers of ISCs, ENTs, GOBs, and PANs. Nuclei are blue. Organoids were stained with OLFM4 (red), lysozyme (LYZ; green), mucin-2 (MUC2; green), sucrase isomaltase (SI; red), or chromogranin A (CHGA; green). 60× magnification; bar = 25 μm. Supporting data sets are deposited in the figshare repository [50]. CHGA chromogranin A, CRY crypt, ENT enterocyte, GOB goblet cell, H&E hematoxylin and eosin stain, HOM homogenates of intestinal mucosa, ISC intestinal stem cell, L lumen, Lgr5 leucine-rich repeat containing G-protein-coupled receptor 5, LYZ lysozyme, MUC2 Mucin-2, OLFM4 Olfactomedin-4, PAN Paneth cell, SI sucrase isomaltase, TYP typical

Bright-field images (Fig. 1a, top row) show the morphological complexity of organoids treated with Wnt and Notch modulators to direct their growth and development. ISC and PAN organoids typically have convoluted and narrow-diameter lumina and numerous buds, just like those of frequently studied TYP organoids (not shown, [20]), which contain disproportionately larger numbers of PANs and ISCs. In contrast, ENT and GOB organoids have larger luminal diameters and fewer, if any, buds. The average luminal diameters of ENT and GOB organoids are similar: 127 ± 17 and 123 ± 14 μm (*n* = 10), respectively. H&E staining of the various organoid types show organoid size and cellular complexity at both 20× (Fig. 1a, middle row) and 60× (bottom row) magnification. Nuclear chromatin, which stains blue to purple (basophilic), suggests a basolateral position of the nuclei in all cell types depicted, as would also be found in vivo. ISC organoids not only displayed a more basophilic nuclear stain, but also had significantly larger nuclei (by almost twofold, *P* < 0.05, Table 1) compared to those of other organoids. Virtually all cells in GOB organoids seemed to have mucus, and each GOB had numerous distinct white unstained granules, seemingly poised for secretion near the apical membrane (Fig. 1a, bottom row). PANs have apical secretory granules that were pink and hence more acidophilic than those of the GOB organoids. ENTs in ENT organoids were columnar, similar to ENTs in vivo. Like those in ISC organoids, the nuclei in PAN organoids were ~33% larger compared to those in ENT organoids and GOB organoids, and occupy a significant amount of cell volume (Table 1; Fig. 1a, bottom row).

**Table 1:** Dimensions of cells in different organoids

Organoid	Nuclei length, μm	Cell length, μm	Cell width, μm
ISC	11.0^a^ ± 0.20	18.5^a^ ± 0.56	4.7^a^ ± 0.23
ENT	5.8^b^ ± 0.22	13.9^b^ ± 0.35	4.8^a^ ± 0.20
GOB	5.2^b^ ± 0.22	19.4^a^ ± 0.76	11.4^b^ ± 0.48
PAN	7.6^c^ ± 0.18	16.6^a^ ± 0.45	5.2^a^ ± 0.43

*ENT* enterocyte, *GOB* goblet cell, *ISC* intestinal stem cell, *PAN* Paneth cell

^a,b,c^*P* < 0.05 within a parameter, *n* = 6 organoids

PAS combined with Alcian blue staining, which indicates the presence of polysaccharides, glycoproteins, and glycolipids, demonstrates the relative paucity of these compounds in lightly stained ISC and ENT organoids (Fig. 1b, left columns). In contrast, there was significant staining of secretory carbohydrate-rich granules in GOBs enriched in GOB organoids. Interestingly, PAN granules stain PAS magenta near the apical membrane, indicating that their secretory granules are enriched in carbohydrate moieties [28]; the basal region occupied mostly by nuclei remained opaque (Fig. 1b, right columns).

Interestingly, ENTs in ENT organoids were slightly shorter compared to cells in ISC, GOB, and PAN organoids (Table 1), while GOBs containing mucus granules in GOB organoids were significantly wider compared to individual cells in ISC, ENT, and PAN organoids. The length and diameter of ENTs in ENT organoids were similar to those of intestinal cells fractionated from the lower villus regions [29]. Previous work has observed that young GOBs near the villus base tended to be much larger, but their volumes decreased toward the upper villus regions, areas where mucin vesicles might already have been secreted [30]. Thus, these directed organoids are morphologically normal despite being exposed to growth factors and growth factor inhibitors, and likely comprise relatively younger cells, as would be expected since these organoids were collected just 2–3 days after differentiation from ISCs was initiated. The lifespan of ENT organoids is 5 days while that of GOB organoids is 4.5 days [20], similar to those of ENTs and GOBs in vivo. In contrast, ISC organoids, after reaching large sizes with numerous buds, can be dispersed and reseeded into new Matrigel, and thus theoretically, they are able to live (be passaged) indefinitely.

#### Biomarker distribution

The comparative expression levels of biomarkers among TYP, ISC, ENT, GOB, and PAN organoids, along with starting crypt material as well as proximal intestinal homogenate, confirmed that directed organoids were highly enriched in specific cell types (Fig. 1c) (*P <* 0.001 for all biomarkers tested). Levels of the stem cell biomarker leucine-rich repeat-containing G-protein coupled receptor 5 (*Lgr5*) were highest in ISC organoids, ~5-fold higher than TYP, which has proportionally a large number of ISCs [20, 21], and 300–500-fold higher than those of ENT and GOB organoids as well as crypt homogenates and intestinal mucosal homogenates. Lysozyme (*Lyz*, a PAN biomarker) was most highly expressed in PAN organoids, and expression levels were ≥ 20-fold higher than those of crypt samples, intestinal homogenates, and other organoids. GOB marker mucin-2 (*Muc2*) was most highly expressed in GOB organoids, about 800-fold higher than in ISCs, and five- to tenfold higher than in other samples. Levels of mRNA of the enteroendocrine biomarker chromogranin A (*Chga*), interestingly, were similar between GOB and PAN organoids, and both were 5- to 25-fold higher compared to those of other organoids, crypts, and homogenates. Expression of the ENT biomarker sucrase isomaltase (SI) was highest in ENT organoids whose expression levels were ~3- to 15-fold greater than in those of other organoids, crypts, and homogenates. Similar results were obtained for alkaline phosphatase, another ENT biomarker (not shown). Finally, the mRNA levels of another stem cell biomarker, *Olfm4*, were greatest in ISC organoids.

Biomarker mRNA expression was confirmed with immunofluorescence of biomarkers in each organoid type (Fig. 1d). An antibody against OLFM4, whose mRNA expression pattern (Fig. 1c) mimics that of *Lgr5*, was used to mark ISCs by immunofluorescence, as antibodies against LGR5 do not seem to work. An additional stem cell biomarker, CD44, was also measured by immunofluorescence, and determined to be abundant in ISC organoids and present in PAN organoids (Additional file 3: Figure S1). OLFM4 was highly visible in the apical region of ISC organoids but was largely absent in ENT and GOB organoids (Fig. 1d). OLFM4 is secreted, and intense immunofluorescence in the closed lumen of ISC organoids reflects accumulated secretions. Its modest accumulation in the lumen of PAN organoids indicate the presence of some stem cells. ENT organoids were lined by large numbers of cells containing significant amounts of SI (~almost 90% SI-positive [20]), some MUC2 (~10%) and, rarely, CHGA. There were virtually no LYZ-positive cells. Virtually all cells in GOB organoids contained MUC2 (~90%), a few SI (15%) and some CHGA (~5%; the total can exceed 100% as some cells co-express two different biomarkers), but, interestingly, virtually no OLFM4 or LYZ. Depending on the plane of focus, some cells that seem devoid of contents may actually contain MUC2 (Additional file 4: Figure S2). PAN organoids had high levels of LYZ (65% [20]) and CHGA (15%), some SI and OLFM4 (~5–10%), but no MUC2. Images of control sections incubated without primary antibodies are depicted in Additional file 4: Figure S2.

In summary, the predominant cell type in ISC organoids is stem cells at levels much higher than estimated in vivo (< 1%). In ENT organoids, the predominant cell type is ENTs at proportions similar to or greater than most published estimates in vivo (~80%). In GOB organoids, the predominant cell type is GOBs at levels much higher than in vivo (< 10%), and in PAN organoids, the predominant cell type is PANs at levels more than in vivo (~5%). As previously noted [21], PAN and GOB organoids both express significant numbers (~15%) of enteroendocrine cells as indicated by its biomarker, CHGA. Unfortunately, generating organoids, particularly secretory, with a single cell type is not yet possible, and we and others [21] have been unable to reduce the levels of enteroendocrine cells in organoids directed to contain primarily PANs or GOBs.

### Tight junction proteins and the leak pathway

#### mRNA expression

**Fig. 2:**
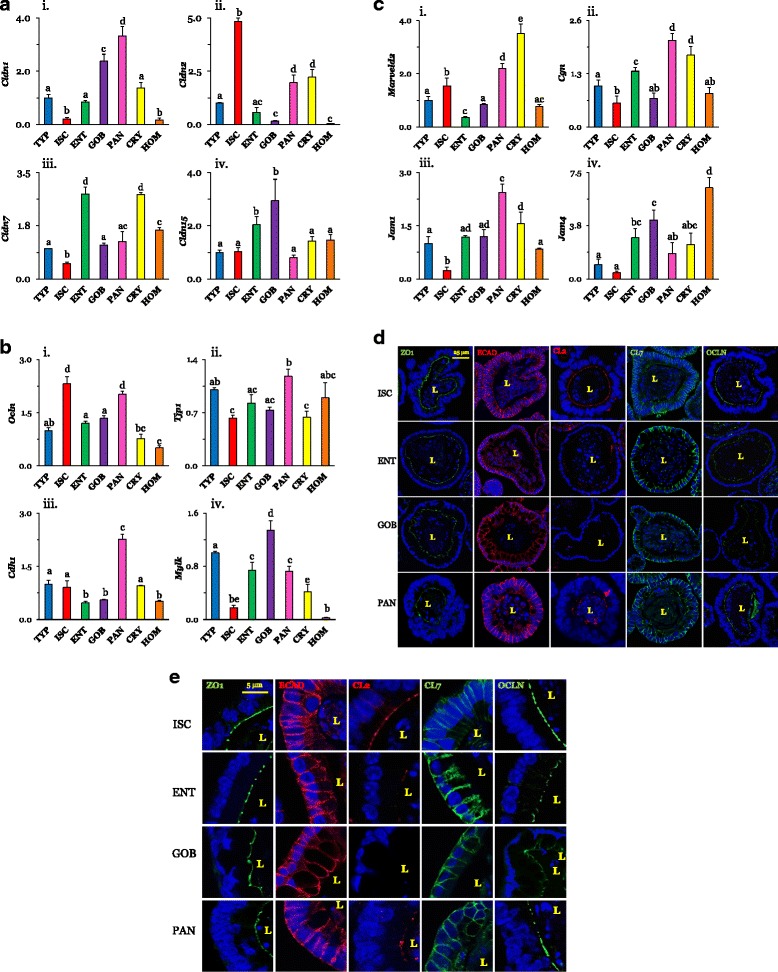
**a** Expression of claudins in different cell types normalized to that in TYP organoids (=1.0). **i** *Cldn1* seemed greatest in GOBs and PANs (*n* = 4 ISC; *n* = 3 others). **ii** *Cldn2* was highest in ISCs (*n* = 4 ISCs; *n* = 3 others). **iii** *Cldn7* was high in ENTs (*n* = 4 ENTs; *n* = 3 others). **iv** *Cldn15* was relatively homogenous (*n* = 4 ENTs; *n* = 3 others). *P*^a,b ,c,d^ ≤ 0.05. **b,c** Expression of TJ proteins in different cell types. **b,i** Occludin (*Ocln*) was higher in ISCs and PANs (*n* = 5 ISCs; *n* = 3 others). **ii** ZO-1 (*Tjp1*) was similar among organoids (*n* = 4 for ISCs, ENTs; *n* = 3 others). **iii** Epithelial cadherin 1 (*Cdh1*) was relatively high in PANs (*n* = 4 ENTs; *n* = 3 others). **iv** Myosin light chain kinase (*Mylk*) was lowest in ISCs (*n* = 3). **c,i** Tricellulin (*Marveld2*) was lowest in ENTs (*n* = 4 TYPs, ISCs, ENTs; *n* = 3 others). **ii** Cingulin (*Cgn*) expression was high in PANs and CRYs (*n* = 5 ENTs; *n* = 3 PANs, CRYs; *n* = 4 others). **iii** Expression of junctional adhesion molecule-1 (*Jam1*) and **iv** JAM4 (*Jam4*) were lowest in ISCs (*n* = 5 ENTs; *n* = 4 TYPs, ISCs, GOBs; *n* = 3 others). **d** Immunofluorescence of representative TJ proteins in organoids probed with ZO-1 (green), ECAD (red), CLDN2 (CL2; red), CLDN7 (CL7; green), or OCLN (green) (60×, bars = 25 μm). **e** Cellular location. ZO-1 staining is mainly in the apical area and ECAD in the basolateral area. CLDN2 is found along the brush border of ISCs and PANs but not in ENTs and GOBs. CLDN7 seems basolateral but expressed less in ISCs. OCLN seems apical. Supporting data sets are deposited in the figshare repository [50]. Cdh or ECAD, E-cadherin, Cgn cingulin, Cldn or CL claudin, CRY crypt, ENT enterocyte, GOB goblet cell, HOM homogenates of intestinal mucosa, ISC intestinal stem cell, Jam, junctional adhesion molecule, L lumen, Marveld2 MARVEL domain containing 2, Mylk myosin light chain kinase, Ocln occludin, PAN Paneth cell, Tjp or ZO1 tight junction protein, TYP typical

Since there are numerous TJ proteins, we focused on ones that have been shown to be expressed significantly in the small intestine, to vary in expression along the crypt–villus axis, or potentially to regulate changes in paracellular permeability [1, 6]. mRNA levels of all organoid claudins were within an order of magnitude of those in isolated crypts or mucosal homogenates, suggesting that expression was similar to that in vivo. Claudin-1 (*Cldn1)* was most highly expressed (*P* < 0.0001 by one-way ANOVA) in secretory GOB and PAN organoids (~3- to 10-fold higher than that in TYP and ENT organoids and least expressed in ISC organoids; Fig. 2a). In contrast, claudin-2 (*Cldn2)* expression was highest in ISC organoids, being ~2.5- to 50-fold higher than that in crypt, mucosal homogenates, and all other organoids (*P* < 0.0001). Claudin-7 (*Cldn7*; Fig. 2a) was highest in CRY and in ENT organoids over all others (*P* < 0.0001). Claudin-15 (*Cldn15*) was the least heterogeneous, but was still most highly expressed in ENT and GOB organoids (*P* < 0.01). Expression of specific claudins in crude mucosal homogenates tended to vary depending on the levels of that claudin in the ENT organoids, reflecting the abundance of ENTs in the mucosa.

Occludin *(Ocln)* expression among organoids tended to vary less than that of claudins (Fig. 2a,b). *Ocln* was expressed approximately twofold more in ISC and PAN organoids than in TYP, ENT, and GOB organoids (*P* < 0.0001). Variation among organoids in *Tjp1* (ZO-1; Fig. 2b) expression was modest (*P* = 0.02), suggesting that ZO-1 expression may be ubiquitous and similar among cell types. There was a marked cell-type dependent variation in E-cadherin (*Cdh1* or ECAD) (*P* < 0.0001) and in myosin light chain kinase (*Mylk*) (*P* < 0.0001). *Mylk* expression was highest in GOB and least in ISC organoids (eightfold less than that in GOBs). PAN organoids had the highest expression of *Cdh1* relative to all other samples (> 2–4-fold).

Tricellulin *(Marveld2)* expression was ~2- to 5-fold higher in ISC and PAN organoids than in TYP, ENT, and GOB organoids (*P* < 0.0001), like that of occludin (Fig. 2c). Variation among organoids in *Cgn* (cingulin) expression was modest (*P* = 0.02). There was also a modest cell-type dependent variation for junctional adhesion molecule-1 (JAM1; *P* = 0.05) and JAM4 levels (*P* < 0.0001), which are both expressed least in ISCs. JAM proteins are found at TJs and participate in the regulation of TJ integrity and permeability, while cingulin interacts with many TJ proteins and modifies their function [4].

In summary, the expression of many important players in the regulation of TJ permeability often varies markedly among different small intestinal cell types.

#### Protein expression and localization

We examined using immunofluorescence the expression of CLDN2 and CLDN7, whose mRNA levels were strongly modulated by cell type, and also the expression of ZO-1, whose deletion disrupts TJs [13], OCLN, which regulates macromolecular TJ permeability [2, 31], and ECAD, a structural protein associated mainly with adherens junctions [1]. ZO-1 was expressed sharply in virtually all cells of ISC, ENT, GOB, and PAN organoids (Fig. 2d,e). ZO-1 staining formed a relatively clear, narrow, punctate line along the apical surface of an organoid (Fig. 2d,e). Likewise, ECAD proteins seemed ubiquitously expressed in all organoid types. In contrast to ZO-1, which was limited primarily to or near the apical membrane, ECAD clearly lined the basolateral membrane regions of all cells in ISC, ENT, GOB, and PAN organoids (Fig. 2d,e). In GOB organoids, ECAD also seemed to be expressed in the apical region.

The distribution of CLDN2 was clearly dependent on cell type. ISC organoids had high amounts of CLDN2 while ENT and PAN organoids had low amounts, and it was not found in GOB organoids. In contrast, CLDN7 seemed to be expressed in all cell types. CLDN7 clearly lined the basolateral membrane areas and was noticeably absent from the apical area of all organoids except ISCs. OCLN was apically oriented and seemed to be greater in ISC and PAN organoids, confirming the pattern of mRNA distribution.

In summary, the expression of OCLN seemed low in GOB organoids, that of CLDN2 was high in ISC organoids, and that of ZO-1 similar in all organoids. CLDN2 was apically oriented in ISC and PAN organoids while ECAD and CLDN7 were largely basolateral, with some apical staining present in certain cell types.

#### Dextran permeability of directed organoids

**Fig. 3:**
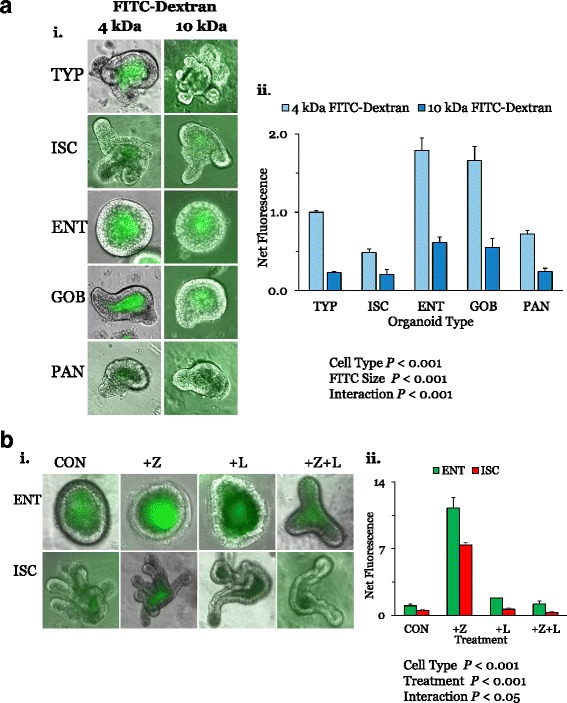
**a** Effects of cell type and dextran size on macromolecular permeability. A serosa to lumen gradient of 1.25 μM 4 or 10 kDa dextran was imposed on TYP, ISC, ENT, GOB, and PAN organoids for 30 min. **i** Representative images clearly depict the higher dextran accumulation in TYP, ENT, and GOB organoids. **ii** Levels of net fluorescence in all organoids were then analyzed as described in the text and normalized to that in 4 kDa TYP organoids (=1.0). (*n* = 4 for ISCs, ENTs, GOBs, and PANs, and *n* = 2 for TYPs, for both 4 and 10 kDa). Using two-way ANOVA, the serosal to luminal flux of both dextran levels was higher in ENT and GOB organoids, and 10 kDa dextran permeability was lower. **b** Effects of AT1002 (an active fragment of ZO toxin) and larazotide on permeability to 4 kDa dextran. ENT and ISC organoids were incubated overnight in 10 μg/mL AT1002 (+Z) or 12.5 mM larazotide acetate (+L) or both (Z + L) and then permeability to 4 kDa dextran was determined (**i**). Using two-way ANOVA, the dextran flux was higher in ENTs and greatest in AT1002-treated organoids (**ii**). (For ENTs, *n* = 3 each for CONs, +Z, +L, and *n* = 2 for + Z + L; for ISCs, *n* = 3 for CONs, and *n* = 2 for + Z, +L and + Z + L). Supporting data sets are deposited in the figshare repository [50]. CON control cell, ENT enterocyte, FITC fluorescein isothiocyanate, GOB goblet cell, ISC intestinal stem cell, PAN Paneth cell, TYP typical

Since the distribution of TJ proteins was heterogeneous, we determined whether the leak pathway would likewise differ between crypt- and villus-dwelling cell types. Using two-way ANOVA, we found highly significant effects for cell type and molecular weight on dextran transport into the organoid lumen (Fig. 3a). We saw that 4 kDa dextran was transported into the lumen of organoids at rates approximately threefold greater than 10 kDa dextran, suggesting that macromolecules with a Stokes radius of 1.4 nm may be better than those of 2.3 nm at distinguishing cell-type differences in the leak pathway of small intestinal TJ. Moreover, 4 kDa dextran accumulated in ENT and GOB lumen at amounts much higher (~2.5- to 4-fold) than those in ISC and PAN organoids. A similar pattern was observed for 10 kDa dextran accumulated fluorescence. Organoids were not permeable to 40 kDa dextrans [24], hence larger molecular weights were not tested. There was a strong statistical interaction, suggesting that cell type affected the effect of molecular size on dextran transport from serosa to lumen.

In summary, the macromolecular flux into ENT and GOB organoids is higher than that into ISC and PAN organoids, suggesting that villus-dwelling cell types may have leak pathways more permeable than those of crypt-dwelling cell types.

#### AT1002 increases dextran permeability of ENT and ISC organoids

We used 4 kDa dextrans to evaluate the effect of AT1002, which is known to disassemble TJ proteins and lead to marked increases in small intestinal permeability, and that of the TJ-regulator larazotide [25]. Larazotide is a novel therapeutic agent targeting TJ regulation in patients with celiac disease [27]. Using two-way ANOVA, we found dramatic effects for cell type and AT1002 treatment (*P* < 0.001). With AT1002, both ENT and ISC organoids exhibited a dramatic increase in 4 kDa dextran permeability (11- and 7-fold, respectively) compared to untreated ENT and ISC organoids (Fig. 3b). Larazotide by itself had no effect on dextran permeability. However, when organoids were incubated in AT1002 in the presence of larazotide, permeability was similar to that of control, untreated levels. Thus, TJ sensitivity to AT1002 in organoids mimics that in vivo, suggesting the similar mechanisms of AT1002 disruption are addressable by larazotide treatment. Although dextran permeabilities are different between ISC and ENT organoids, these were similarly susceptible to AT1002.

#### Dextran accumulation over time

**Fig. 4:**
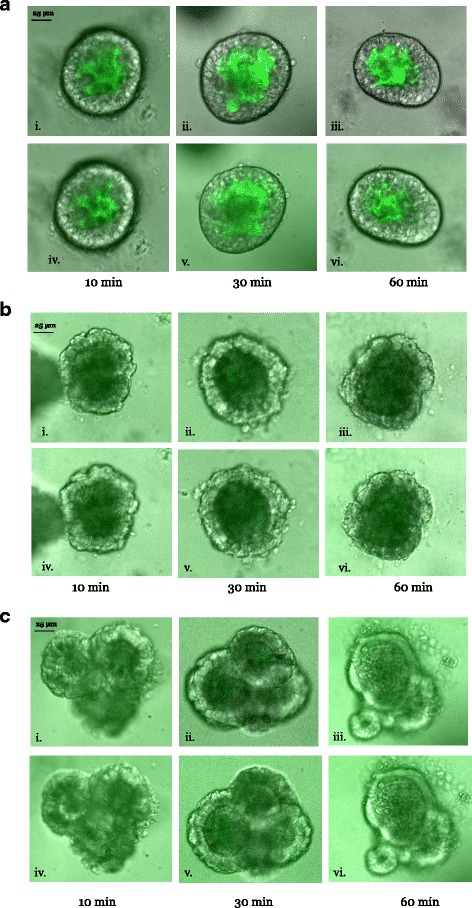
Dextran accumulation in ENT, PAN, and ISC organoids over time. ENT, PAN, and ISC organoids were each exposed to 4 kDa dextran for 10, 30, and 60 min, washed, and then imaged with an inverted confocal DMi8 microscope equipped with a CSU-W1 spinning disk (see text). Then, 5-mm Z-stacks were acquired above and below the vertical midpoint of the organoid. Representative images from above (**i, ii, iii**) and below (**iv, v, vi**) the midpoint of ENT (**a**), PAN (**b**), and ISC (**c**) organoids at 10, 30, and 60 min intervals (*n* = 5 or 6 organoids). Dextran accumulation was clearly greater in ENT organoids at all time points. Luminal dextran levels increased with incubation time. Supporting data sets are deposited in the figshare repository [50]. ENT enterocyte, ISC intestinal stem cell, PAN Paneth cell

After 10 min of incubation, ENT organoids had accumulated significant amounts of 4 kDa dextran (Fig. 4a). Dextran levels were similar throughout the organoid lumen, as images from Z-stacks of the same organoid exhibited similar fluorescence intensities relative to the background. Similar observations were made for 5 or 6 ENT organoids. Relative to the 10-min incubation, dextran levels increased by 68 ± 12% after 30 (*n* = 6) and by 65 ± 10% after 60 (*n* = 6) min of incubation. When incubated over the same duration, PAN (Fig. 4b) and ISC (Fig. 4c) organoids accumulated much less dextran, confirming the results in Fig. 3a. The paucity of dextran levels was evident throughout the lumen of the numerous PAN and ISC organoids examined, even after 60 (Fig. 4b,c) and 80 min (not shown). Since similarly shaped and sized ENT and PAN organoids accumulated dextrans at vastly different rates, while differently shaped and sized PAN and ISC organoids both accumulated dextrans at similarly limited rates, these findings also suggest that the shapes of organoids likely do not affect the accumulation of dextran, and by extension, of our estimates of permeability of the leak pathway.

### Dedifferentiation and TJ plasticity

#### Morphometrics and biomarker expression

Since cell type and thus, differentiation from stem to secretory and absorptive progenies affected TJ protein levels and permeability, we devised a method to dedifferentiate mature cell types [20]. We assessed the plasticity of TJ protein expression and dextran permeability in differentiated ENT organoids, and examined whether the acquisition of ISC-like biomarkers in dedifferentiated ENT organoids is accompanied by a return to an ISC-like TJ composition and permeability.

**Fig. 5:**
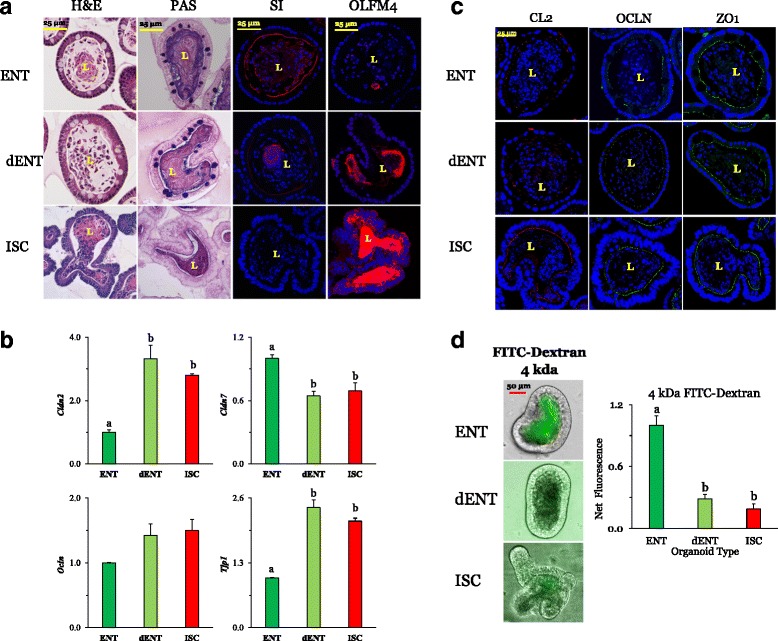
Effect of dedifferentiation of ENT organoids on expression of biomarker immunofluorescence and histological staining. From ISC precursors, ENT organoids were cultured for 3 days with C59 + valproic acid to full differentiation as indicated by the peak expression of biomarkers. To force dedifferentiation, the Wnt inhibitor C59 was removed from the medium, and ENT organoids were then exposed for 36 h to 6 μM CHIR (dENT). **a** ENT and dENT organoids were stained with H&E as well as PAS. The morphology of and cell dimensions within dENT organoids were similar to those of ENT organoids. However, the immunofluorescence of the biomarkers indicated there was a significant loss of ENT-marker sucrase isomaltase (SI) staining in dENT organoids as well as an increase in secretion of stem cell marker OLFM4. **b** The expression of TJ mRNA in dENT organoids. *Cldn2* increased with dedifferentiation (*n* = 4 for ENTs, *n* = 3 for dENTs and ISCs). *Cldn7* decreased slightly with dedifferentiation (*n* = 4), while *Ocln* levels did not change (*n* = 5 for ENTs, *n* = 3 for dENTs, and *n* = 4 for ISCs). *Tjp1* (ZO-1) tended to increase (*P* = 0.09) with dedifferentiation (*n* = 5 for ENTs, *n* = 4 for dENTs, and *n* = 3 for ISCs). *P*^a,b,c^ ≤ 0.05. **c** Immunofluorescence staining of CLDN2, OCLN, and ZO-1 in dedifferentiated cells. Nuclei are stained blue. Organoids were stained with the TJ protein CLDN2 (CL2; red), OCLN (green), and ZO-1 (green). Representative organoids are shown at 60× magnification. Bars are 25 μm. **d** Functional changes due to dedifferentiation. 4 kDa dextran permeability decreased in dENT organoids, *P*^a,b,c^ ≤ 0.05 (*n* = 4 for ENTs, 3 for dENTs, and 5 for ISCs). Supporting data sets are deposited in the figshare repository [50]. Cldn claudin, dENT dedifferentiated enterocyte, ENT enterocyte, FITC fluorescein isothiocyanate, H&E hematoxylin and eosin stain, ISC intestinal stem cell, L lumen, Ocln occludin, OLFM4 olfactomedin-4, PAS periodic acid-Schiff stain, SI, sucrase isomaltase, TJ tight junction, Tjp or ZO-1 tight junction protein

H&E and PAS staining of ENT and dENT organoids showed no remarkable morphological (Fig. 5a) or morphometric changes caused by dedifferentiation, compared to those depicted in Fig. 1a,b and Table 1, which show marked differences between ENT and ISC organoids. The percentage of GOBs in ENT organoids (~15% [20]) was similar to that of dENT organoids (14.7 ± 0.8, *n* = 6). The cell length was similar between ENT (Table 1) and dENT (14.0 ± 0.5 μm, *n* = 6) organoids. Despite similar morphometrics and histological morphology, ENT and dENT organoids exhibited marked differences in levels of the ENT biomarker SI and the stem cell biomarker OLFM4. SI staining in dENTs was significantly reduced compared to that of ENT organoids (Fig. 5a). In fact, only the lumen (likely containing cells exfoliated prior to dedifferentiation) had SI immunoreactivity while the ISC organoids had no SI at all, as would be expected. In contrast, OLFM4 levels were almost nonexistent in ENT organoids but then staining increased markedly in the dENT lumen where OLFM4 was secreted, indicating that organoids were indeed in the process of dedifferentiation, and were beginning to look the same as ISCs. Moreover, mRNA expression of stem cell biomarkers increased while that of ENT biomarkers decreased from ENT to dENT organoids [20]. Thus, while ENT and dENT organoids shared similar structure and cell composition, the activation of Wnt (via CHIR) and of Notch (via VPA) pathways induced the loss of markers of differentiation and the reacquisition of markers of stemness, as previously shown by us.

#### TJ protein expression

Previously observed differences in mRNA expression of *Cldn2*, *Cldn7*, *Ocln*, and *Tjp1* between ENTs and ISCs in Fig. 2a,b were similar to the pattern of differences between ENTs and dENTs (Fig. 5b). Expression of *Cldn2* (*P* < 0.001), *Ocln* (*P* < 0.20), and *Tjp1* (*P* < 0.0001) in dENT organoids increased from ENT levels and became similar to those of ISC organoids, while expression of *Cldn7* decreased to ISC levels (*P* < 0.005). Thus, mRNA expression of these TJ components changed during dedifferentiation from that of ENTs to dENTs, to reflect ISC levels.

#### Immunocytochemistry

CLDN2 was present in ISC but not ENT organoids (Fig. 5c), as shown previously (Fig. 2d,e). CLDN2, however, was not highly expressed in the apical membrane of dENT organoids and thus, it did not follow the pattern of changes in *Cldn2* mRNA. OCLN and ZO-1 were expressed along the apical membrane in a punctate manner in both ISC and ENT organoids as before, and were also expressed in dENTs.

#### Dextran permeability

Relative to ISC organoids, ENT organoids were more permeable (Fig. 5d) to 4 kDa dextran as previously observed (Fig. 3a). dENT organoids had a 70% reduction (*P* < 0.001) in permeability compared to ENT organoids, so that dENT permeability became similar to that of ISC organoids. This reduction in dextran permeability back to ISC levels suggests TJs exhibit functional plasticity, which may correlate with changes in the expression or location of unidentified TJ proteins whose levels or location become similar to those of ISC organoids. Here, similarly shaped and sized ENT and dENT organoids have markedly different levels of luminal dextran, suggesting that dextran accumulation is not significantly affected by organoid form.

### Effect of time after induction of differentiation from ISC

**Fig. 6:**
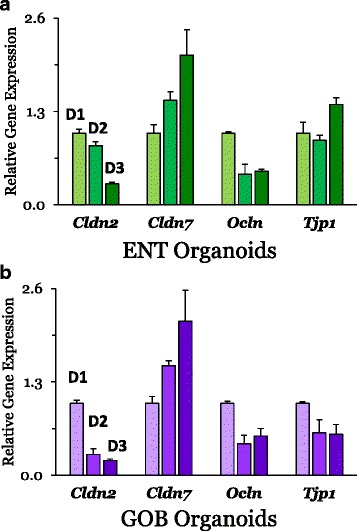
Effect of time after directed differentiation from ISC on mRNA expression of TJ proteins from ENT (**a**) and GOB (**b**) organoids. Within each gene, all treatments are normalized to day 1 (D1) post-differentiation for both ENT and GOB organoids. Data were analyzed using one-way ANOVA (see text for *P* values). **a** *Cldn2* (*n* = 3 for ENTs D1 to D3), *Cldn7* (*n* = 3 for D1 and *n* = 2 for D2 as well as D3), *Ocln* (*n* = 3 for D1 as well as D3, and *n* = 2 for D2), and *Tjp1* (ZO-1) (*n* = 3 for all). **b** *Cldn2* (*n* = 3 for GOBs D1 to D3), *Cldn7* (*n* = 3 for D1 and *n* = 2 for D2 as well as D3), *Ocln* (*n* = 3 for all), and *Tjp1* (ZO-1) (*n* = 3 for GOB D1 as well as D3, and *n* = 2 for D2). Trends in mRNA expression of TJ proteins during directed differentiation from ISC to ENT or GOB organoids matched those depicted in Fig. 2a,b. Supporting data sets are deposited in the figshare repository [50]. Cldn claudin, D day, ENT enterocyte, GOB goblet cell, ISC intestinal stem cell, Ocln occludin, TJ tight junction, Tjp or ZO-1 tight junction protein

Relative to the effects of cell type, the effects of organoid age or time after induction of differentiation from ISCs were much more modest. In ENT organoids, TJ-related genes were altered due to post-differentiation time and generally reflect a transition from ISC to ENT cell-type composition, which stabilized, based on the time course of biomarker expression, 2–3 days after transition from ISCs [20]. *Cldn2* (*P* < 0.0001) and *Ocln* (*P* < 0.002) decreased significantly over time, while *Cldn7* (*P* < 0.05) increased by twofold (*P* < 0.01). Recall that *Cldn7* expression is typically low in ISCs. There was a modest effect (*P* = 0.05) of post-differentiation time on *Tjp1* expression (Fig. 6a).

In GOB organoids, TJ-related genes were also altered due to the post-differentiation time. *Cldn2* (*P* < 0.0001) and *Ocln* (*P* < 0.025) expression decreased significantly by day 3 of differentiation to GOB organoids (Fig. 6b). In contrast, *Cldn7* expression increased (*P* = 0.025), while that of *Tjp1* did not change (*P* = 0.10). When differentiation is triggered from ISC to ENT or GOB organoids, the post-differentiation time course would reflect the in vivo journey of postmitotic cells from villus base to tip, and migration would be accompanied by generally small changes in expression of genes coding for TJ proteins. In fact, claudin differentiation may result in several physiologically distinct TJs within the lifetime of the same cell [14].

## Discussion

### Macromolecular permeability of tight junctions depends on cell type

Previous steric estimates have calculated that small intestinal crypts would allow the paracellular flux of 5–6 nm macromolecules and would be far leakier than the TJ of cells lining the upper villus, which were predicted to restrict flux only to macromolecules <0.6 nm [19]. However, our results suggest that TJs of ENTs and GOBs lining the villus column may actually have leak pathways more permeable to 1–2 nm macromolecules than those of ISCs and PANs. Since regulation of the pore may be independent of that of the leak pathway [32], this finding does not imply that ion permeability is also greater in the TJs of ENTs and GOBs.

ZO-1, occludin, and tricellulin regulate the leak pathway [2–5, 33]. Occludin, tricellulin (MarvelD2), and MarvelD3 belong to one protein family and they have distinct but overlapping functions at the TJ. Occludin was initially considered not to be essential for TJs because transepithelial electrical resistance is similar between occludin-KO and wild-type mice [13], but this may be due to the pore pathway being independent of occludin. Occludin-KO mice, however, are growth-retarded and their gastrointestinal tract is chronically inflamed. In fact, dephosphorylation [34], inhibition [35], and knockdown [36] of occludin were observed to increase, while overexpression would prevent increases in paracellular permeability of the leak pathway during inflammation [2, 31]. Occludin-depleted intestinal epithelial monolayers had markedly enhanced TJ permeability to large molecules, which could be modeled by size-selective channels with radii of ~6.25 nm [37]. Finally, synthetic peptides with the same sequence as occludin extracellular loops disrupt TJs and increase paracellular permeability [38, 39], but it is not clear whether these peptides affected the pore or leak pathways.

Tricellulin is normally localized at tricellular TJs with much less expression at bicellular TJs. Overexpression of tricellulin reduces macromolecular permeability without affecting ion permeability [40, 41], suggesting that tricellulin at tricellular TJs may be the site of the leak pathway. After occludin knockdown, tricellulin is redistributed to occupy bicellular TJs also [37], indicating that tricellulin may partially compensate for occludin loss. In turn, tricellulin loss may result in occludin redistribution near tricellular contact points [42], although most other studies did not have similar observations [43–45]. Our findings that occludin and tricellulin expression are greater in ISC and PAN organoids are indeed consistent with observations that their TJs are less permeable to dextran. Marveld3 is in TJs but there is little evidence it directly regulates permeability [46], and it cannot restore function if occludin or tricellulin is deleted [47].

ZO-1, which interacts with claudins, F-actins, and occludin, is essential for TJ and cytoskeletal structure [1, 4], and thus, it is well represented and located apically in all cell types. ZO-1 expression does not seem to change during differentiation from stem cell to ENT or GOB. This homogeneity suggests that under normal conditions, ZO-1 may not regulate differences in TJ macromolecular permeability. In several disease and injury states, ZO-1 is often redistributed away from the cell membranes, along with occludin, leading to increases in intestinal permeability.

Clues regarding potential cell-type differences in junctional structure emerged only in the 1980s with observations via freeze fracture microscopy of marked differences in microanatomy and in lanthanum/barium reactivity of the paracellular spaces between GOBs and absorptive cells [48]. This anatomical heterogeneity of TJ between cell types was followed by estimates predicting functional heterogeneity. Either the paracellular pathways in villus regions would be less permeable than those in the crypt [19] or these pathways in the crypt regions would be more permeable than those in the villus [18]. Until recently, these contradictory predictions could not be assessed experimentally for lack of an appropriate methodology. Our findings using directed organoids clearly show that TJs of crypt-dwelling ISCs and PANs seem less permeable to uncharged macromolecules compared to those of ENTs and GOBs, as predicted by Madara and colleagues [18].

### TJ disruption and larazotide

ZO toxin opens TJs by binding to PAR2 (proteinase activated receptor 2) and EGFR (epidermal growth factor receptor) [25] in the apical membrane of ENTs. The response of nonenterocytes to this toxin has never been studied. We observed that AT1002, though introduced in the medium bathing the basolateral surface of all organoids, still disrupted TJs, leading to the markedly increased paracellular transport of dextran in both ENT- and ISC-enriched organoids. Thus, AT1002 disrupts TJs by mechanisms that have similar effects on different cell types. The mechanism by which AT1002 disrupts TJs in the basolateral compartment is unknown; perhaps, a long overnight incubation would allow this small peptide to access the lumen.

Larazotide acetate has previously been shown to reduce the high intestinal permeability caused by gliadin in celiac disease, by preventing disruption of TJs [26, 27]. Larazotide is a small octapeptide, which, even when administered from the serosal compartment, can go through the paracellular pathway during an overnight incubation, to affect TJs luminally. Larazotide by itself had no effect on the normal permeability of ISC and ENT organoids, suggesting it likely did not alter the natural arrangement of components of normal TJs. However, by completely blocking the AT1002-induced increase in permeability in both ISC and ENT organoids, its mode of action is likely independent of cell type.

### TJ expression during differentiation and dedifferentiation

Differentiation and cell type clearly affect the distribution of certain TJ proteins. While the crypt–villus location of claudins-2 and -7 have been established in classical histological sections [1, 6], it would have been difficult to distinguish clearly their presence in specific cell types. Here, we show claudin-2 to be uniquely present in both ISC and PAN organoids, and claudin-7 to be in differentiated cells, particularly ENTs (Table 2). Likewise, differences in occludin distribution would have been quite difficult to depict in traditional histological presentations because of the overwhelming difference in the numbers of different cell types, but here we are able to show that ISC and PAN organoids have different levels of occludin from those of ENT and GOB organoids (Table 2).

**Table 2:** Relative permeability of organoids as well as mRNA and protein expression of TJ proteins

Organoid Permeability		Cldn2		Cldn7		Ocln		ZO1		Cdh1	
		mRNA	Protein	mRNA	Protein	mRNA	Protein	mRNA	Protein	mRNA	Protein
ISC	**+**	**++++**	**++++**	**+**	**++**	**++++**	**++**	**+++**	**+++**	**+++**	**++++**
ENT	**++++**	**++**	**+**	**++++**	**++++**	**+++**	**+**	**+++**	**++**	**++**	**+++**
GOB	**++++**	**+**	**-**	**++**	**+++**	**+++**	**-**	**+++**	**++**	**++**	**++++**
PAN	**+**	**+++**	**++**	**+++**	**++++**	**++++**	**+++**	**++++**	**+**	**++++**	**++++**

- = no expression, + = low, ++ = modest, +++ = high, ++++ = very high expression

*Cldn* claudin, *ENT* enterocyte, *GOB* goblet cell, *ISC* intestinal stem cell, *PAN* Paneth cell, *TJ* tight junction, *ZO-1* tight junction protein-1

The reduction of paracellular permeability in and the changes in the TJ composition of dENT organoids suggest that there is inherent plasticity in the expression of TJ proteins and in the permeability of the paracellular pathway. Thus, directed organoids from mouse genetic models without specific TJ protein(s) would be excellent in identifying those that regulate differentiation-related alterations in TJ permeability. This is important because intestinal tumorigenesis is initiated by dedifferentiation of mature cells and their partial acquisition of stem-cell-like properties, likely resulting in changes in TJ properties. Changes in the expression of TJ molecules have been observed to result in key changes in the TJ barrier function, which can lead to the successful metastasis of a number of different cancer types [49].

### Limitations

These directed organoids contain other cell types that could potentially confound our findings. However, each organoid type is much more highly enriched in a specific cell compared to in situ populations in the small intestine, providing us with an experimental approach to estimate TJ heterogeneity among different cell types, an objective that would be difficult to pursue in vivo and even in vitro as isolated intestinal cells have extremely short lifespans (~1 h) [29]. Since we cannot control the cell types that will be adjoined, the properties of TJs between different cell types cannot be evaluated using this experimental method. Under physiological conditions in vivo, nonenterocytes are often surrounded by ENTs, thus a major limitation of this study is that we are only able to show the macromolecular permeability of large clusters of the same cell type. It may be important to investigate the composition of the nonenterocyte–ENT TJs, which may differ substantially from TJs between nonenterocytes. The confounding effects of lumen shape and size, as well as of luminal contents, on macromolecular permeability are important considerations and should be investigated further. The pharmacological approaches used to direct differentiation may also affect TJ expression, independent of the differentiation process itself. Unfortunately, alternative approaches affecting other components of Wnt and Notch signaling have yet to produce viable, directed organoids [21].

## Conclusions

The main finding in our study is that different intestinal cell types exhibit marked differences in the kinds and levels of TJ proteins, resulting in significant variations in macromolecular permeability among cell types (Table 2). Highly conserved TJs are present in various tissues of, and many TJ proteins are shared by, all vertebrates, whose epithelia are also composed of many cell types. Thus, our findings are relevant for vertebrates that rely on permselective epithelia not only to obtain valuable compounds but also to prevent the entry of harmful substances from the environment.

## Additional files

Additional file 1: Table S1. Primer sequences. (DOC 39 kb)

Additional file 2: Table S2. Antibody information. (DOC 32 kb)

Additional file 3: Figure S1. Immunofluorescence of the stem cell marker CD44 in organoids enriched in stem cells (ISCs), enterocytes (ENTs), goblet cells (GOBs), and Paneth cells (PANs). CD44 staining is shown in red, while nuclei are blue. Notice the intense staining of CD44-positive cells in ISC organoids. There was virtually no CD44 in ENT and GOB organoids. Modest levels of CD44 were located in PAN organoids where some stem cells are found. (PDF 351 kb)

Additional file 4: Figure S2. Top row: Immunofluorescence image of MUC2 showing the same organoid with different focus levels to show that goblet cells may appear filled with mucus or appear empty depending on the focus level (arrows). Bottom row: Images depicting organoid paraffinized sections incubated with the secondary antibody only, to show potential autofluorescence in the green (488) or red (546) spectrum. (PDF 755 kb)

## Abbreviations

C59: 4-(2-Methyl-4-pyridinyl)-N-[4-(3-pyridinyl)phenyl]benzeneacetamide; porcupine inhibitor
CCM: Crypt culture medium
Cdh1 or ECAD: E-cadherin, Epithelial cadherin
Cgn: Cingulin
CHIR: 6-[[2-[[4-(2,4-Dichlorophenyl)-5-(5-methyl-1H-imidazol-2-yl)-2-pyrimidinyl]amino]ethyl]amino]-3-pyridinecarbonitrile; inhibitor of glycogen synthase kinase 3
CHGA: Chromogranin A
Cldn1: Claudin-1
Cldn15: Claudin-15
Cldn2 or CL2: Claudin-2
Cldn7 or CL7: Claudin-7
CON: Control
CRY: Crypt
DAPT: N-[(3,5-Difluorophenyl)acetyl]-L-alanyl-2-phenyl]glycine-1,1-dimethylethyl ester; γ-secretase inhibitor
dENT: Dedifferentiated enterocyte
EGF: Epidermal growth factor
ENT: Enterocyte
FITC: Fluorescein isothiocyanate
GOB: Goblet cell
H&E: Hematoxylin and eosin stain
HOM: Homogenates of intestinal mucosa
ISC: Intestinal stem cell
JAM1: Junctional adhesion molecule 1
JAM4: Junctional adhesion molecule 4
Lgr5: Leucine-rich repeat containing G-protein-coupled receptor 5
LYZ: Lysozyme
Marveld2: MARVEL domain containing 2
MUC2: Mucin-2
Mylk: Myosin light chain kinase
Ocln or OCLN: Occludin
OLFM4: Olfactomedin-4
PAN: Paneth cell
PAS: Periodic acid-Schiff stain
PBS: Phosphate buffered saline
SI: Sucrase isomaltase
TJ: Tight junction
Tjp1 or ZO-1: Tight junction protein-1, also known as zonula occludens
TYP: Typical cell
VPA: Valproic acid

## References

[1] Shen L, Weber CR, Raleigh DR, Yu D, Turner JR (2011). Tight junction pore and leak pathways: a dynamic duo. Annu Rev Physiol..

[2] Odenwald MA, Turner JR. The intestinal epithelial barrier: a therapeutic target? Nat Rev Gastroenterol Hepatol. 2016;14(1):9-21. 10.1038/nrgastro.2016.169 PMC5554468 27848962

[3] Zihni C, Mills C, Matter K, Balda MS (2016). Tight junctions: from simple barriers to multifunctional molecular gates. Nat Rev Mol Cell Biol.

[4] Suzuki T (2013). Regulation of intestinal epithelial permeability by tight junctions. Cell Mol Life Sci.

[5] Lechuga S, Ivanov AI (2017). Disruption of the epithelial barrier during intestinal inflammation: quest for new molecules and mechanisms. Biochim Biophys Acta.

[6] Capaldo CT, Nusrat A (2015). Claudin switching: physiological plasticity of the tight junction. Semin Cell Dev Biol..

[7] O'Brien P, Corpe CP (2016). Acute effects of sugars and artificial sweeteners on small intestinal sugar transport: a study using Caco-2 cells as an in vitro model of the human enterocyte. PLoS One.

[8] Meunier V, Bourrie M, Berger Y, Fabre G (1995). The human intestinal epithelial cell line Caco-2; pharmacological and pharmacokinetic applications. Cell Biol Toxicol.

[9] Cheng H, Leblond CP (1974). Origin, differentiation and renewal of the four main epithelial cell types in the mouse small intestine. V. Unitarian theory of the origin of the four epithelial cell types. Am J Anat.

[10] Montgomery RK, Breault DT (2008). Small intestinal stem cell markers. J Anat.

[11] Umar S (2010). Intestinal stem cells. Curr Gastroenterol Rep.

[12] Gerbe F, van Es JH, Makrini L, Brulin B, Mellitzer G, Robine S (2011). Distinct ATOH1 and Neurog3 requirements define tuft cells as a new secretory cell type in the intestinal epithelium. J Cell Biol.

[13] Furuse M (2009). Knockout animals and natural mutations as experimental and diagnostic tool for studying tight junction functions in vivo. Biochim Biophys Acta.

[14] Gunzel D, Yu AS (2013). Claudins and the modulation of tight junction permeability. Physiol Rev.

[15] Gunzel D, Fromm M (2012). Claudins and other tight junction proteins. Compr Physiol.

[16] Holmes JL, Van Itallie CM, Rasmussen JE, Anderson JM (2006). Claudin profiling in the mouse during postnatal intestinal development and along the gastrointestinal tract reveals complex expression patterns. Gene Expr Patterns.

[17] Rosenthal R, Gunzel D, Krug SM, Schulzke JD, Fromm M, Yu AS (2017). Claudin-2-mediated cation and water transport share a common pore. Acta Physiol.

[18] Marcial MA, Carlson SL, Madara JL (1984). Partitioning of paracellular conductance along the ileal crypt–villus axis: a hypothesis based on structural analysis with detailed consideration of tight junction structure–function relationships. J Membr Biol.

[19] Fihn BM, Sjoqvist A, Jodal M (2000). Permeability of the rat small intestinal epithelium along the villus–crypt axis: effects of glucose transport. Gastroenterology.

[20] Kishida K, Pearce SC, Yu S, Gao N, Ferraris RP (2017). Nutrient sensing by absorptive and secretory progenies of small intestinal stem cells. Am J Physiol Gastrointest Liver Physiol.

[21] Yin X, Farin HF, van Es JH, Clevers H, Langer R, Karp JM (2014). Niche-independent high-purity cultures of Lgr5(+) intestinal stem cells and their progeny. Nature Methods.

[22] Pearce SC, Mani V, Boddicker RL, Johnson JS, Weber TE, Ross JW (2013). Heat stress reduces intestinal barrier integrity and favors intestinal glucose transport in growing pigs. PLoS One.

[23] Armstrong JK, Wenby RB, Meiselman HJ, Fisher TC (2004). The hydrodynamic radii of macromolecules and their effect on red blood cell aggregation. Biophys J.

[24] Zietek T, Rath E, Haller D, Daniel H (2015). Intestinal organoids for assessing nutrient transport, sensing and incretin secretion. Sci Rep..

[25] Fasano A (2011). Zonulin and its regulation of intestinal barrier function: the biological door to inflammation, autoimmunity, and cancer. Physiol Rev.

[26] Gopalakrishnan S, Durai M, Kitchens K, Tamiz AP, Somerville R, Ginski M (2012). Larazotide acetate regulates epithelial tight junctions in vitro and in vivo. Peptides.

[27] Khaleghi S, Ju JM, Lamba A, Murray JA (2016). The potential utility of tight junction regulation in celiac disease: focus on larazotide acetate. Therap Adv Gastroenterol.

[28] Mantani Y, Yuasa H, Nishida M, Takahara E, Omotehara T, Udayanga KG (2014). Peculiar composition of epithelial cells in follicle-associated intestinal crypts of Peyer's patches in the rat small intestine. J Vet Med Sci.

[29] Ferraris RP, Villenas SA, Diamond J (1992). Regulation of brush-border enzyme activities and enterocyte migration rates in mouse small intestine. Am J Physiol.

[30] Specian RD, Oliver MG (1991). Functional biology of intestinal goblet cells. Am J Physiol.

[31] Marchiando AM, Shen L, Graham WV, Weber CR, Schwarz BT, Austin JR (2010). Caveolin-1-dependent occludin endocytosis is required for TNF-induced tight junction regulation in vivo. J Cell Biol.

[32] Lingaraju A, Long TM, Wang Y, Austin JR, Turner JR (2015). Conceptual barriers to understanding physical barriers. Semin Cell Dev Biol..

[33] Liang GH, Weber CR (2014). Molecular aspects of tight junction barrier function. Curr Opin Pharmacol..

[34] Jain S, Suzuki T, Seth A, Samak G, Rao R (2011). Protein kinase Cζ phosphorylates occludin and promotes assembly of epithelial tight junctions. Biochem J.

[35] Wong V, Gumbiner BM (1997). A synthetic peptide corresponding to the extracellular domain of occludin perturbs the tight junction permeability barrier. J Cell Biol.

[36] Al-Sadi R, Khatib K, Guo S, Ye D, Youssef M, Ma T (2011). Occludin regulates macromolecule flux across the intestinal epithelial tight junction barrier. Am J Physiol Gastrointest Liver Physiol.

[37] Buschmann MM, Shen L, Rajapakse H, Raleigh DR, Wang Y, Wang Y (2013). Occludin OCEL-domain interactions are required for maintenance and regulation of the tight junction barrier to macromolecular flux. Mol Biol Cell.

[38] Lacaz-Vieira F, Jaeger MM, Farshori P, Kachar B (1999). Small synthetic peptides homologous to segments of the first external loop of occludin impair tight junction resealing. J Membr Biol.

[39] Tavelin S, Hashimoto K, Malkinson J, Lazorova L, Toth I, Artursson P (2003). A new principle for tight junction modulation based on occludin peptides. Mol Pharmacol.

[40] Krug SM, Amasheh S, Richter JF, Milatz S, Gunzel D, Westphal JK (2009). Tricellulin forms a barrier to macromolecules in tricellular tight junctions without affecting ion permeability. Mol Biol Cell.

[41] Westphal JK, Dorfel MJ, Krug SM, Cording JD, Piontek J, Blasig IE (2010). Tricellulin forms homomeric and heteromeric tight junctional complexes. Cell Mol Life Sci.

[42] Ikenouchi J, Furuse M, Furuse K, Sasaki H, Tsukita S, Tsukita S (2005). Tricellulin constitutes a novel barrier at tricellular contacts of epithelial cells. J Cell Biol.

[43] Kamitani T, Sakaguchi H, Tamura A, Miyashita T, Yamazaki Y, Tokumasu R (2015). Deletion of tricellulin causes progressive hearing loss associated with degeneration of cochlear hair cells. Sci Rep..

[44] Nayak G, Lee SI, Yousaf R, Edelmann SE, Trincot C, Van Itallie CM (2013). Tricellulin deficiency affects tight junction architecture and cochlear hair cells. J Clin Invest.

[45] Van Itallie CM, Fanning AS, Holmes J, Anderson JM (2010). Occludin is required for cytokine-induced regulation of tight junction barriers. J Cell Sci.

[46] Steed E, Elbediwy A, Vacca B, Dupasquier S, Hemkemeyer SA, Suddason T (2014). MarvelD3 couples tight junctions to the MEKK1-JNK pathway to regulate cell behavior and survival. J Cell Biol.

[47] Raleigh DR, Marchiando AM, Zhang Y, Shen L, Sasaki H, Wang Y (2010). Tight junction-associated MARVEL proteins marveld3, tricellulin, and occludin have distinct but overlapping functions. Mol Biol Cell.

[48] Madara JL, Trier JS (1982). Structure and permeability of goblet cell tight junctions in rat small intestine. J Membr Biol.

[49] Martin TA, Jiang WG (2009). Loss of tight junction barrier function and its role in cancer metastasis. Biochim Biophys Acta.

[50] Pearce SC, Al-Jawadi A, Kishida K, Yu S, Hu M, Fritzky LF, et al. Marked differences in tight junction composition and macromolecular permeability among different intestinal cell types. 2018. 10.6084/m9.figshare.5729571 10.1186/s12915-018-0481-z PMC5793346 29391007

