# Supplementary material for: Marked differences in tight junction composition and macromolecular permeability among different intestinal cell types
Source: BMC Biol. 2018 Feb 1;16:19. doi: 10.1186/s12915-018-0481-z (PMC5793346; doi:10.1186/s12915-018-0481-z)
Supplement: Supplementary file 1 — Primer sequences. (DOC 39 kb) [file 12915_2018_481_MOESM1_ESM.doc]

Additional file 1: **TableS1**. Primer Sequences

| **Gene Name** | **Sense (5’- 3’) - Forward** | **Antisense (5’- 3’) - Reverse** |
| --- | --- | --- |
| Actin (*Actb*) | TTGTTACCAACTGGGACGACATGG | CTGGGGTGTTGAAGGTCTCAAACA |
| Alkaline Phosphatase, intestinal (*Alpi*) | CTCATCTCCAACATGGAC | TGCTTAGCACTTTCACGG |
| Cadherin-1 (*Cdh1*) | CACCTGGAGAGAGGCCATGT | TGGGAAACATGAGCAGCTCT |
| Chromogranin A (*Chga*) | CGATCCAGAAAGATGATGGTC | CGGAAGCCTCTGTCTTTCC |
| Claudin-1 (*Cldn1*) | TCTACGAGGGACTGTGGATG | TCAGATTCAGCAAGGAGTCG |
| Claudin-2 (*Cldn2*) | GCAAACAGGCTCCGAAGATACT | GAGATGATGCCCAAGTACAGAG |
| Claudin-7 (*Cldn7*) | GGGAGATGACAAAGCGAAGA | CAGAAGGACCAGAGCAGACC |
| Claudin-15 (*Cldn15*) | GCTTCTTCATGTCAGCCCTG | TTCTTGGAGAGATCCATGTTGC |
| Cingulin (*Cgn*) | GACAGTTCTGCAGTCCACC | TAGCTGGTCCTTCTGGTCGT |
| Junctional Adhesion Molecule 1 *(JAM1)* | AGCCGGGAGGAAACTGTTGT | GCAGGTCAATTTGATGGACTCGT |
| Junctional Adhesion Molecule 4 *(JAM4)* | CCACCATGACAGACACTTGG | TAACCAAATGGTGGTGCTGA |
| Leucine-Rich Repeat Containing G Protein- Coupled Receptor 5 *(Lgr5)* | TGAGCGGGACCTTGAAGATT | AGGTGCTCACAGGGCTTGAA |
| Lysozyme (*Lyz*) | ATGGCTACCGTGGTGTCAAG | CGGTCTCCACGGTTGTAGTT |
| Marveld2 | ACGCACCGGGTGTGAAA | TGAACACTCCACACCATCGG |
| Mucin-2 (*Muc2*) | CTTCTGTGCCACCCTCGT | TTCGGGATCTGGCTTCTT |
| Myosin-light Chain Kinase (*Mylk*) | ACATGCTACTGAGTGGCCTCTCT | GGCAGACAGGACATTGTTTAAGG |
| Olfactomedin 4 (*Olfm4*) | GCCACTTTCCAATTTCAC | GAGCCTCTTCTCATACAC |
| Occludin (*Ocln*) | ATCCACCTATCACTTCAGA | TAATCTCCCACCATCCTC |
| Sucrase Isomaltase (*Si*) | ATCCAGGTTCGAAGGAGAAGCACT | TTCGCTTGAATGCTGTGTGTTCCG |
| Zonula Occludin-1 (ZO-1) | GCTAAGAGCACAGCAATGGA | GCATGTTCAACGTTATCCAT |
