# Supplementary material for: Marked differences in tight junction composition and macromolecular permeability among different intestinal cell types
Source: BMC Biol. 2018 Feb 1;16:19. doi: 10.1186/s12915-018-0481-z (PMC5793346; doi:10.1186/s12915-018-0481-z)
Supplement: Supplementary file 2 — Antibody information. (DOC 32 kb) [file 12915_2018_481_MOESM2_ESM.doc]

Additional file 2: **Table S2**. Antibody Information

| **Protein** | **Company** | **Cat #, RRID, lot number** | **Dilution** |
| --- | --- | --- | --- |
| CD44 Antigen (CD44) | BioLegend | 103002, AB_312953, B176899 | 1:200 |
| Chromogranin A (CHGA) | Santa Cruz Biotechnology | sc-13090, AB_2080982, F3014 | 1:200 |
| Claudin-2 (CL2) | Abcam | Ab53032, AB_869174, GR387796-12 | 1:100 |
| Claudin-7 (CL7) | Life Technologies | 34-9100, AB_2533190, QA214025 | 1:100 |
| E-cadherin (ECAD) | BD Transduction Laboratories | 610181, AB_397580, 5274693 | 1:100 |
| Lysozyme (LYZ) | Biogenex | AR024, none, AR0240316 | none |
| Mucin-2 (MUC2) | Santa Cruz Biotechnology | sc-15334, AB_2146667, K0315 | 1:200 |
| Occludin (OCLN) | Life Technologies | 331500, AB_2533101, QD215073 | 1:1000 |
| Olfactomedin-4 (OLFM4) | Cell Signaling | D6Y5A, AB_2650511, 1 | 1:1000 |
| Sucrase Isomaltase (SI) | Santa Cruz Biotechnology | sc-27603, AB_2188721, J0914 | 1:500 |
| Zonula Occludens-1 (ZO-1) | Santa Cruz Biotechnology | SC-33725, AB_628459, F0316 | 1:100 |
