# Supplementary figures and images for: Marked differences in tight junction composition and macromolecular permeability among different intestinal cell types
Source: BMC Biol. 2018 Feb 1;16:19. doi: 10.1186/s12915-018-0481-z (PMC5793346; doi:10.1186/s12915-018-0481-z)

***Supplemental Fig. S1.***

ISC

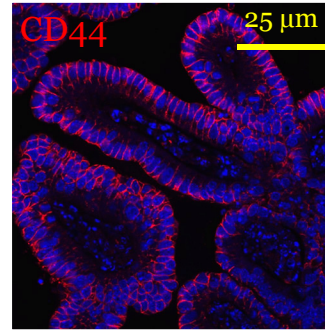

ENT

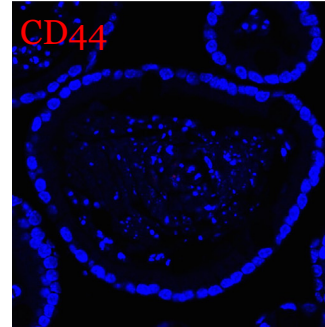

GOB

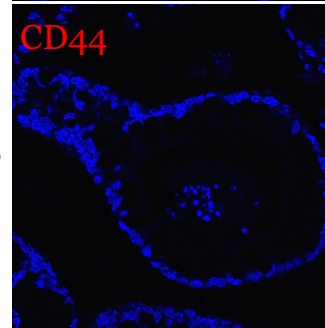

PAN

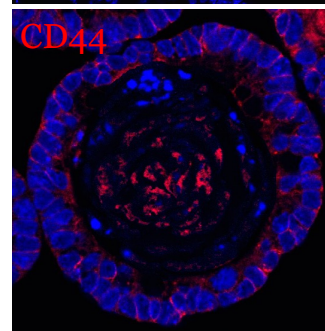

Supplement: Supplementary file 3 — Immunofluorescence of the stem cell marker CD44 in organoids enriched in stem cells (ISCs), enterocytes (ENTs), goblet cells (GOBs), and Paneth cells (PANs). CD44 staining is shown in red, while nuclei are blue. Notice the intense staining of CD44-positive cells in ISC organoids. There was virtually no CD44 in ENT and GOB organoids. Modest levels of CD44 were located in PAN organoids where some stem cells are found. (PDF 351 kb) [file 12915_2018_481_MOESM3_ESM.pdf]

*Supplemental Fig. S2.*

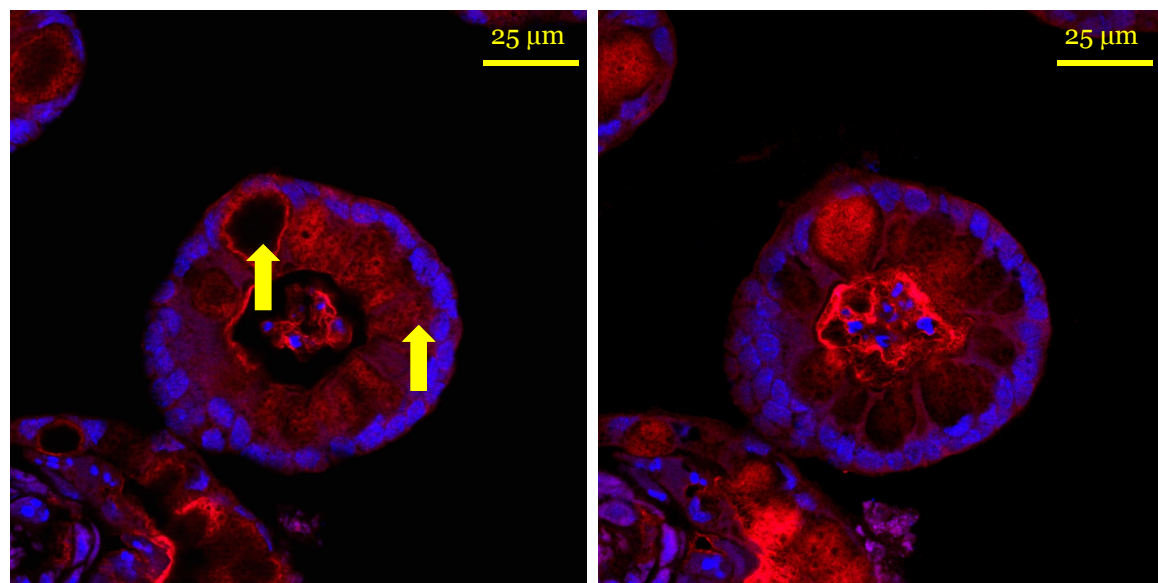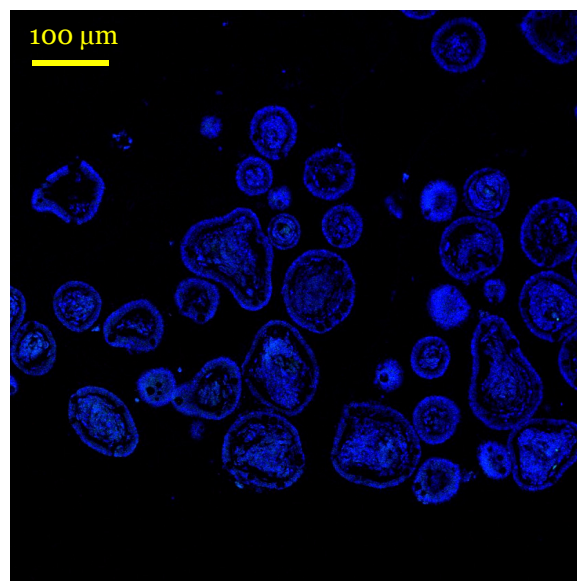

488 Secondary

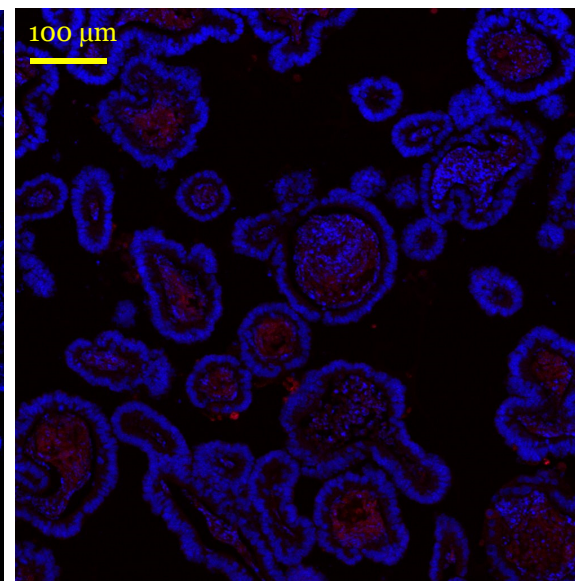

546 Secondary

Supplement: Supplementary file 4 — Top row: Immunofluorescence image of MUC2 showing the same organoid with different focus levels to show that goblet cells may appear filled with mucus or appear empty depending on the focus level (arrows). Bottom row: Images depicting organoid paraffinized sections incubated with the secondary antibody only, to show potential autofluorescence in the green (488) or red (546) spectrum. (PDF 755 kb) [file 12915_2018_481_MOESM4_ESM.pdf]
